# 22q13 deletion syndrome: communication disorder or autism? Evidence from a specific clinical and neurophysiological phenotype

**DOI:** 10.1038/s41398-018-0212-9

**Published:** 2018-08-08

**Authors:** Laura Ponson, Marie Gomot, Romuald Blanc, Catherine Barthelemy, Sylvie Roux, Arnold Munnich, Serge Romana, Nadia Aguillon-Hernandez, Valérie Malan, Frédérique Bonnet-Brilhault

**Affiliations:** 1 UMR 1253, iBrain, Université de Tours, Inserm, Tours, France; 2 Centre Universitaire de Pédopsychiatrie, CHRU de Tours, Tours, France; 3 Université Paris Descartes, Sorbonne Paris Cité, Institut de Psychologie, Laboratoire de Psychopathologie et Processus de Santé (EA 4057), Paris, France; 4 Université Paris Descartes, Sorbonne Paris Cité, Paris, France; 5 Laboratory of Molecular and Pathophysiological Bases of Cognitive Disorders, INSERM UMR 1163, Imagine Institute, Necker-Enfants Malades Hospital, Paris, France; 6 Service d’Histologie-Embryologie et Cytogénétique, Hôpital Necker-Enfants Malades, AP-HP, Paris, France

## Abstract

Phelan–McDermid syndrome is related to terminal 22q13 deletions of various sizes affecting the *SHANK3* gene. In this neurodevelopmental disorder, behavioural symptoms of autism spectrum disorder (ASD) are reported in half of cases. Extensive clinical and neurophysiological characterization is lacking to understand the genotype–phenotype correlation. Eighteen patients (8 males, mean age 12.7 years, SD = 9.2) with known 22q13 deletions were fully explored with determination of deletion size, along with behavioural, language and cognitive standardized assessments. Neurophysiological indices previously reported to be altered in autism (i.e., eye tracking in a social/non-social task and auditory evoked potential mismatch) were also recorded. Thirty-nine percent met ASD clinical criteria, exceeding cut-off scores on both ADI-R (Autism Diagnosis Interview based on the period spanning 4–5 years of age) and ADOS-2 (Autism Diagnosis Observation Schedule for the current period). All patients had intellectual disability and language disability. Deletion size was significantly correlated with expressive and receptive language disability but not with ASD standardized assessment scores. Developmental Quotient tended to be lower in patients with the largest deletions. Using Eye Tracking, smaller pupil size, which is typically described in ASD, was not observed in these patients. Furthermore, atypical shortened latency of mismatch negativity response previously reported in ASD was not observed, whereas the N250 pattern, related to language, was affected. Language disability combined with cognitive deficits may lead to autistic behavioural symptoms, but with different neurophysiological networks compared to typical autism. These results highlight the indication for early speech therapy rather than intensive autism programme to treat these patients.

## Introduction

22q13 deletion syndrome, also known as Phelan–McDermid syndrome (PMS), is a neurodevelopmental disorder characterized by hypotonia, global developmental delay with intellectual disability of varying degrees, severely delayed or absent speech and minor dysmorphia. Autism spectrum disorder (ASD) features are often reported for more than one in two patients but estimates of ASD rates vary significantly across studies depending on the assessment tool used^1^. 22q13 syndrome is the result of a *de novo* or inherited chromosome abnormality, which disrupt *SHANK3*. *SHANK3* haploinsufficiency (occurring through intragenic deletion or point mutation) is enough to cause the neurobehavioral symptoms. This gene encodes for SH3 and multiple ankyrin repeat domains 3, also known as proline-rich synapse-associated protein 2, a multidomain postsynaptic scaffolding protein^2,3^. It has a role in synaptogenesis, in synaptic plasticity and in the regulation of dendritic spine morphology^4,5^. Found in about 0.5% of patients with ASD diagnosis, *SHANK3* has been set as a strong candidate gene for autism^6,7^. Previous studies have found few genotype–phenotype correlations, agreeing to say that larger deletions were associated with increased levels of dysmorphic features, language delay and medical comorbidities^1,8,9^. Among previous clinical studies evaluating the prevalence of ASD in PMS, rates vary from 0% to 94%^10^. This large heterogeneity is mainly explained by the various clinical procedures used, from single phone parental interview to direct behavioural evaluation with standardized assessment. Only one study reported Genotype/ASD phenotype correlation with size deletion with direct behavioural standardized evaluation and this concerned only the first dimension of ASD, i.e., social communication^1^.

Neurophysiological endophenotypes are known to be relevant for genotype/phenotype correlation studies. Regarding ASD research, atypical mismatch negativity of auditory evoked potential (MMN) has been proposed as a candidate endophenotype^11^, distinguishing autism from intellectual disablity^12^. Another component of auditory evoked potential (i.e., N250 amplitude component) has been reported to be correlated with language abilities^13^. Smaller mean pupil size during visual scanning with eye-tracking technology (recorded at baseline and during the presentation of human faces and objects) has also been reported as a potential biomarker in autism and could discriminate children with autism from mental age-matched and chronological age-matched controls^14^.

Combining behavioural, dimensional and neurophysiological explorations, the global aim of our study was to better characterize autistic behaviours in PMS and to test whether these symptoms are related to atypical information processing reported in autism. Furthermore, we aim to correlate the clinical manifestations of the PMS with 22qter deletion sizes.

## Materials and methods

### Subjects

Eighteen patients with known 22q13 terminal deletions were enroled in an institutional review board-approved project with parents providing informed consent for participation. Assent was obtained from the patient when possible. Patients ranged in age from 4 to 37 years (mean 12.7, SD = 9.2). Children and adults (eight males) were fully explored with standardized behavioural, language and cognitive assessments. Neurophysiological indices previously reported to be altered in autism (i.e., eye tracking in a social/non-social task and evoked auditory mismatch potentials) were also recorded. In parallel to these clinical and neurophysiological investigations carried out in the child psychiatry department of the University Hospital of Tours, cytogenetic analyses were conducted in the Cytogenetics Department of the Necker Enfants Malades Hospital in Paris. All participants, or their respective parents, gave written informed consent according to institutional guidelines. The experiment was approved by an institutional review board and conformed to the Code of Ethics of the World Medical Association (according to the Ethical Principles for Medical Research Involving Human Subjects in the Declaration of Helsinki in 2008).

### Clinical assessments

- Psychiatric evaluations using DSM-IV criteria^15^ were conducted by psychiatrists and focused on the assessment of pervasive developmental disorder
- Autism Diagnostic Interview–Revised (ADI-R)^16^, an investigator-based semi-structured instrument, was administered by a trained interviewer to parents. It was used to distinguish autistic disorder from non-autistic pervasive developmental disorder
- Autism Diagnostic Observation Schedule-Generic (ADOS-G)^17^, a direct semi-structured assessment, was used to assess the presence of autism features (in communication, reciprocal social interaction and repetitive or restricted behaviour domains). Trained clinicians administered ADOS-G Module 1 or 3 according to patient age and developmental level.
- The Childhood Autism Rating Scale (CARS)^18^ was use to provide an estimate of autism severity.
- The Behavioural Summarized Evaluation (BSE)^19^ focused on factor 1, which comprises the 13 most relevant items for autism diagnosis.
- Repetitive and Restricted Behaviour Scale (RRBS)^20^.
  Factor 1 (F1) is the sum of 11 items assessing “sensorimotor stereotypies”.
  Factor 2 (F2) is the sum of seven items assessing “reaction to change”.
  Factor 3 (F3) is the sum of eight items assessing “restricted behaviours”.
  Factor 4 (F4) is the sum of seven items assessing “modulation insufficiency”.
  Each item was evaluated according to a five-level Likert scale (0 = “the behaviour is never expressed by the person”, 1 = “weakly expressed”, 2 = “moderately expressed”, 3 = “severely expressed” and 4 = “the behaviour is very characteristic of the person and very severely expressed”).
- Cognitive testing was conducted by psychologists. Tests were selected based on patient age and developmental level to provide a mental age estimate. The Social Cognitive Evaluation Battery^21^ and Differential scale of intellectual efficiency revised form^22^ were used. Developmental quotients (DQs) were calculated using chronological age and mental age estimates.
- Language testing was conducted by a speech therapist: expressive language was rated from 0 to 5. (Clinically corresponding to: 0 = No language_ 1 = Babbling_ 2 = “Isolated or juxtaposed words” _ 3 = “Simple sentences” _ 4 = “Embedded or coordinated sentences” _ 5 = “Complex language”). Receptive language was rated from 0 to 5. Clinically corresponding to: 0 = No understanding_ 1 = Contextual understanding_ 2 = Lexical understanding_ 3 = Simple sentences understanding_ 4 = Embedded or coordinated sentences understanding_ 5 = Conversational understanding

### Electrophysiological recordings

#### Eye tracking

The stimuli battery, the eye-tracking procedure and the method of measuring the pupil are the same as those previously described in detail elsewhere^14^. Visual stimuli included ten neutral faces (photographs of humans aged 18–35 years) on a beige background and ten objects from daily life on a beige background. Each image was harmonized in terms of colours, background, position and face size. Each of the 20 visual stimuli was successively projected on a 21 inch computer screen for 4 s with an inter-stimulus interval of 0.5 s consisting of a blank black slide. Gaze was checked to be centred in the middle of the screen between each stimulus. There was no instruction before or during the experiment. Participants sat in a comfortable armchair, in the darkness (the brightness of the room was 2 lx during the dark inter-stimulus screens and 10 lx during the stimuli), 90 cm from the computer screen. We recorded gaze direction with a FaceLab eye-tracking system (60 Hz frequency). This instrument measured the time spent exploring the entire screen in both “face screen” and “object screen” conditions. The two measures were the mean duration per each 4-s exposure time for each condition. We did not exclude any participants for these two measures and compared this time spent exploring with the expected norm for age. For each stimulus category, mean pupil size was computed every 0.250 s (then plotted over time to obtain a pupil waveform) for each of the three stimulus categories. For each stimulus, slide pupil data were inspected, in order to eliminate artefacts (blinking and loss of tracking), which were corrected using linear interpolation when they were no longer than 350 ms. We used pupil traces at least 500 ms in length with artefacts that were no longer than 400 ms or no more than 20% of trace duration. We also computed a difference score (difference between each value obtained every 0.250 s and value at t = 0 s) to show the change in pupil size from baseline every 0.250 s (*t* = 0), in order to obtain relative pupil dilation. Finally, pupillary dilation was obtained by averaging the relative pupil dilation values over the last 2 s of presentation of the stimulus.

#### Electroencephalography

Auditory stimulus sequences consisted of 1000 Hz standard tones and 1100 Hz deviant tones (probability of occurrence: *p* = 0.15) delivered in random order, with the constraint that each deviant tone was preceded by at least three standard tones. All tones were delivered at an intensity of 70 dB sound pressure level for a duration of 50 ms (5 ms rise/fall). A block of 1000 stimuli was presented binaurally through headphones with a constant stimulus onset asynchrony of 700 ms. Subjects watched a silent movie on a TV screen during the recording session that lasted 15 min. The whole experiment was controlled by a Neuroscan electroencephalography (EEG) system (Synapse amplifier, Scan 4.3 and Stim2 software).

EEG data were recorded from nine Ag/AgCl electrodes referenced to the nose Fz, Cz, Pz, C3, C4, T3, T4, M1, and M2. The impedance value of each electrode was less than 10 kΩ. The ELAN software package was used for analysis and visualization of EEG and event-related potentials (ERPs)^23^. The EEG and electrooculogram were amplified and filtered with an analogue bandpass filter (0.3–70 Hz). Movement artefacts were discarded manually and automatic correction of the deviations due to ocular activity was applied, based on a spatial filter transform. The analysis period was 700 ms (sampling rate: 500 Hz) including a 100 ms prestimulus baseline. A zero phase-shift low-pass filter (30 Hz) was then applied to ERPs.

The event-related potentials to deviant tones included at least 120 responses for each subject. MMN was measured in the difference waveforms obtained by subtracting the responses to the standard tones from responses to the deviant stimuli. MMN and N250 peak amplitude and latency were measured in each subject by locating the most negative deflection within a ± 50 ms latency window around the peak of the grand average waveform of the corresponding age group.

### Genetic analysis

A customized 60 K oligonucleotide microarray (Agilent Technologies, Santa Clara, CA) was used for this study according to the manufacturer’s recommendations. The microarray was spotted using 60,000 oligonucleotides corresponding to sequences across the whole genome (60,000 probes with a space of 60 kb between 2 consecutive probes). The 22q13.3 region was enriched in oligonucleotides with an average distance between two probes of 10 kb, in order to precisely define the deletion breakpoints. Genomic positions are relative to human genome Build NCBI37/hg19 (Table 1).

**Table 1:** Summary of the 22q13 deletions characterized by array CGH

Patient number	Genomic coordinates	Chromosomal region	Size of the deletion (Mb)
1	arr[GRCh37] 22q13.2.q13.33 (42982735 × 243006204_51193680 × 1)	22q13.2.q13.33	8.187
1	arr[GRCh37] 22q13.2 q13.33 (43454452 × 2,43484726_51193680 × 1)	22q13.2q13.33	7.708
1	arr[GRCh37] 22q13.31q13.33 (44889875 × 2,45002243_51193680 × 1)	22q13.31q13.33	6.191
1	arr[GRCh37] 22q13.33 (46547478 × 246591347–51193680 × 1)	22q13.33	4.602
1	arr[GRCh37] 22q13.31q13.33 (46988921 × 247046025_51,193,680 × 1)	22q13.31q13.33	4.147
1	arr[GRCh37] 22q13.31q13.33 (47244365 × 2,47308036_51193680 × 1)	22q13.31q13.33	3.885
1	arr[GRCh37] 22q13.32 q13.33 (48538897 × 2,48600735_51193680 × 1)	22q13.32q13.33	2.592
1	arr[GRCh37] 22q13.32q13.33 (49286818 × 2,49371757 × 1)	22q13.32q13.33	1.80
1	arr[GRCh37] 22q13.33(49647323 × 24975283451,149,235 × 1,51,151,912 × 2)	22q13.33	1.396
1	arr[GRCh37] 22q13.33 (50131,773 × 2,50,172,917_51,193,680 × 1)	22q13.33	1.020
1	arr[GRCh37] 22q13.33 (51123491_51304566 × 1)	22q13.33	0.181
4	arr[GRCh37] 22q13.33 (51121513 × 2,51122452–51193680 × 1)	22q13.33	0.071
1	arr[GRCh37] 22q13.33 (51,121,513 × 2,51,122,452 × 1)	22q13.33	0.056
1	arr[GRCh37] 22q13.33(51115076 × 2,51116,12851,145299 × 1,51,146,403 × 2)	22q13.33	0.029

Genomic coordinates are indicated according to the ISCN (International System for Human Cytogenomic Nomenclature) 2016. *CGH* comparative genomic hybridization

### Data analysis

Descriptive statistics were calculated across all measures and organized according to phenotypic domains: ASD features, DQ, receptive and expressive language. For BSE, only the most relevant factor was analysed, F1, as detailed above.

Standards responses in neurophysiology were considered by age group (4–7 years, 8–11 years, 12–14 years, 15–18 years, adults for auditory evoked potentials and 5–10 years, 11–14 years, 15–20 years and 21 years and older for eye tracking).

Statistical analyses were performed using the Statistica v10 software (Statsoft, Inc). Nonparametric tests were used (i.e., Spearman’s rank correlation tests). In all cases, tests were performed on the two-sided 5% level of significance. Adjustment was made for multiple testing using Bonferroni correction.

## Results

### Clinical findings

Phenotypic data were available for all participants with rates of 100% completion on diagnostic and cognitive evaluations. All had intellectual disability and language disability. Except one patient who had a 55 QD score, they all had mild to profound intellectual disability with an average DQ of 25.4 [10–55] (mean [range]). Language disability was broadly severe with expressive language disability of 1.1 [0–4] (mean [range]) corresponding to no language or isolated words at best; and receptive language disability of 1.6 [0–4] (mean [range]) corresponding to no understanding or contextual and lexical understanding. According to expectations, ASD clinical features reported by parents through ADI-R exceeded cut-off scores in 56% of cases. Seventy-two percent met social interaction domain and communication domain criteria, and 61% met repetitive behaviour and restricted domain criteria.

Using ADOS-2, 50% met ASD clinical criteria exceeding cut-off scores. However, only 39% remained positive when both ADOS-2 (Autism Diagnosis Observation Schedule for current period) and ADI-R were required.

In average, CARS scores were under the 30 cut-off for mild autism (28.1 ± 7.6 (mean ± SD)) just as BSE F1 scores were under the 27 cut-off for autism (24.1 ± 10.4 (mean ± SD)).

Repetitive and restricted behaviour scale (RRBS) scores were very low with F1: 4.7 [0–13] (mean [range]), while the maximum score is 44; F2: 0.8 [0–4] (mean [range]), while the maximum score is 28; F3 5.4 [0–17] (mean [range]), while the maximum score is 32; and F4 4.8 [0–17] (mean [range]), while the maximum score is 28. Considering RRBS ranges, we observed that even the highest individual scores remained very low, indicating very few repetitive and restricted behaviours. Thirteen patients had a 0 score for Factor 2, which has been shown to be the most specific for autism^20^. Two others had 1 and the remaining three had 4. As a sum of seven items rated from 0 to 4 and considering that a score of 1 means 1 out of 12 and can only correspond to a single “weakly expressed” item, RRBS F2 was only clearly observed in three out of eighteen patients.

As expected, we observed a strong correlation between DQ scores and expressive (*R* = 0.73; *N* = 18; *p* = 0.0006***) and receptive (*R* = 0.74; *N* = 18; *p* = 0.0005***) language disability scores. These results remained significant after Bonferroni correction.

### Genetic findings

Chromosomal microarray analysis (CMA) was conducted on seventeen patients. The deletion ranged in size from 29 kb to 8.1 Mb (Table 1). No additional pathogenic copy number variation (CNV) was detected in these patients. In one case, CMA could not be performed. This patient had been included for clinical and neurophysiological explorations but not in genotype–phenotype analysis.

### Electrophysiological findings

#### Eye tracking

**Fig. 1:**
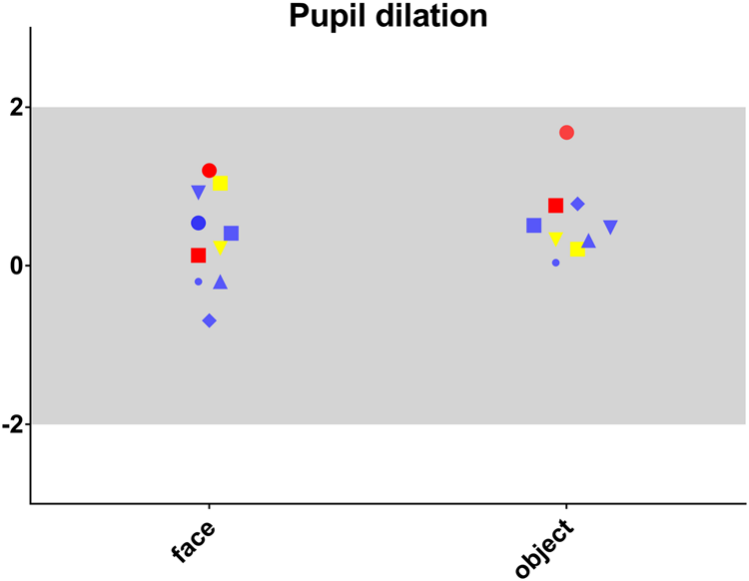
Individual results for pupil dilation measurements. Pupil dilation in standard deviation by age (SDA) is presented for each patient in face and object conditions. Patient results are colour coded as follows: Red for criteria exceeding cut-off scores for both ADI-R and ADOS-2; yellow when meeting only ADI-R criteria and blue when criteria do not exceed both cut-off scores

Thirteen patients underwent eye-tracking analysis and the results were interpretable for nine and eight of them under face and object conditions respectively. The results per patient are shown in Fig. 1. For each condition the mean time spent exploring was measured for all participants ((mean ± SD) Face 3.34 s ± 0.18; Object 3.10 s ± 0.25). One-sample Student *t*-tests (T) were applied to analyse standardized data (*z*-scores), showing that this time did not differ between each patient and the expected norm, either for age, face (*t* = − 2.23; df = 8; *p* = ns) or object (*t* = − 1.30; df = 7; *p* = ns) conditions.

Changes in pupil size when the stimuli were faces ((mean ± SD) − 0.300 ± 0.329) were in the norm for age (*t* = 1.30; df = 8; *p* = ns). Pupil sizes observed when the stimuli were objects ((mean ± SD) − 0.309 ± 0.320) were greater (*t* = 4.70; df = 7; *p* = 0.002), although they remained within the limits of 2SD.

#### Auditory evoked potentials

**Fig. 2:**
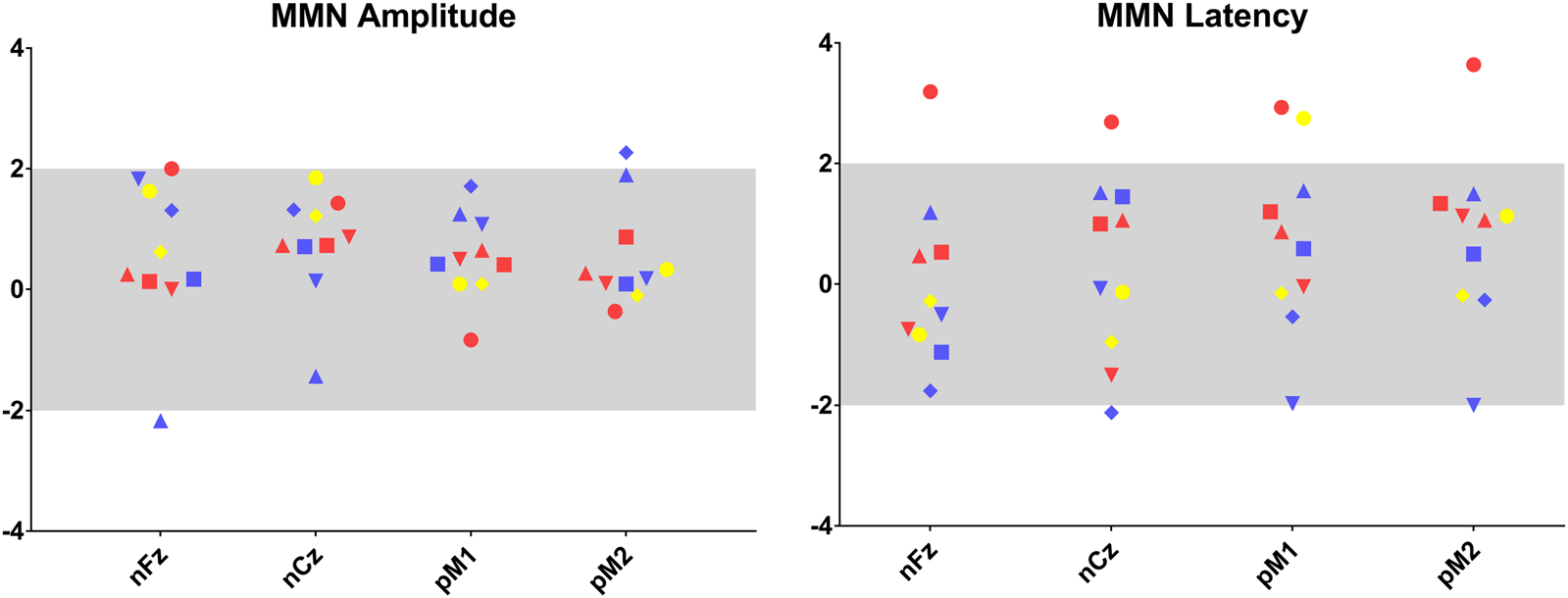
Individual results for auditory evoked potential measurements. Amplitude and latency in standard deviation by age (SDA) are presented for each patient, for MMN in nFz, nCz, pM1 and pM2 positions. As in Fig. 1, results colour coded according ASD assessment profiles

**Fig. 3:**
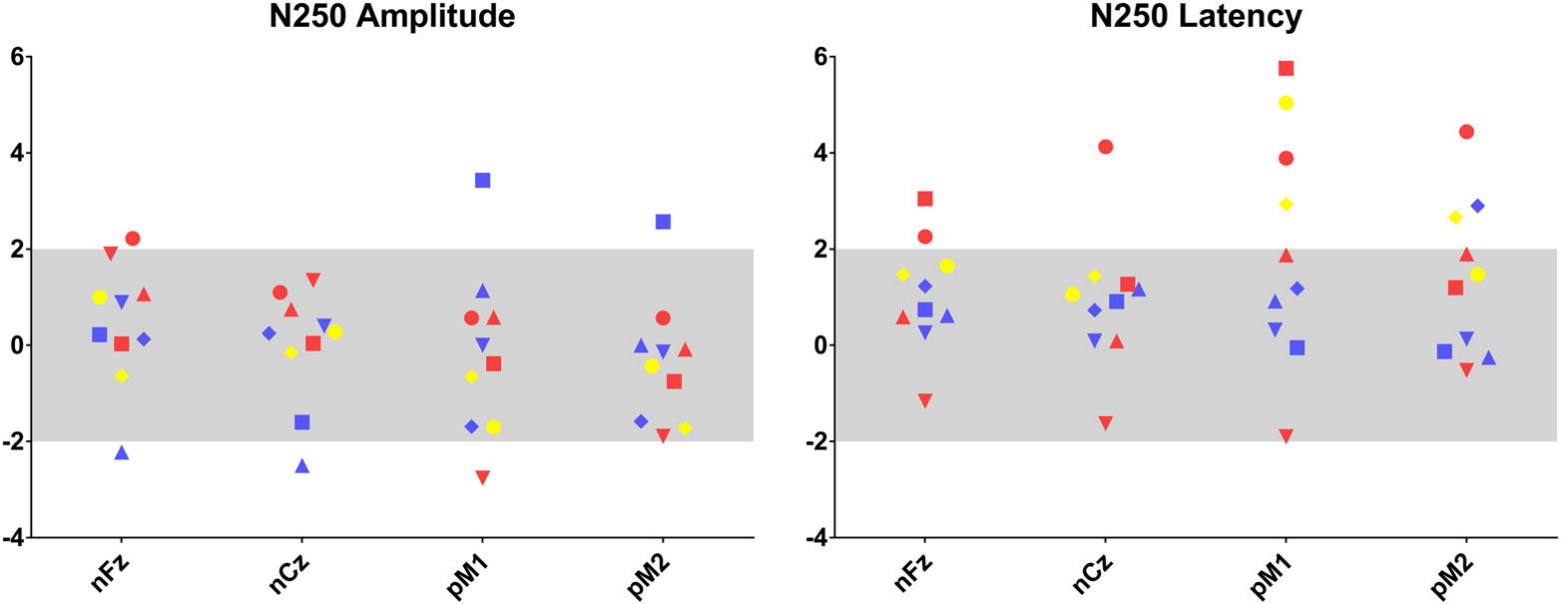
Individual results for auditory evoked potential measurements. Amplitude and latency in SD by age (SDA) are presented for each patient, for N250 in nFz, nCz, pM1 and pM2 positions. As in Fig. 1, results colour coded according ASD assessment profiles

Ten patients underwent auditory evoked potentials. Individual results are shown in Figs. 2 and 3.

N250 was recorded for all patients over fronto-central sites, inverting in polarity at mastoid electrodes. Given our sample size, we did not perform statistical analyses of the electrophysiological responses by subgroup according to the ADI/ADOS profiles. Thanks to colour code however, visual inspection showed (Fig. 3) that most of the patients meeting the ADI ± ADOS criteria for autism had longer N250 latency compared to the norm for age ((mean ± SD) Fz 280 ms ± 36; Cz 280 ms ± 42; M1 284 ms ± 59; M2 275 ms ± 48). For all patients, the N250 amplitude ((mean ± SD) Fz − 3.4μV ± 2.4; Cz − 3.4μV ± 1.7; M1 3.0 μV ± 2.1; M2 2.6 μV ± 1.5) was in the norm for age ± 2 SD.

MMN at Fz and Cz were identified for all patients, with the positive counterpart at mastoid electrodes. MMN peak latency was globally in the norm for age (Fz 172 ms ± 38; Cz 164 ms ± 37; M1 194 ms ± 41 and M2 198 ms ± 42). Only one patient was above the ± 2 SD threshold at both Fz and Cz sites, and presented longer latencies. As presented in Fig. 2, the patients with ADI + /ADOS + assessments did not display the shorter MMN latencies typically described in ASD populations. MMN amplitude at Fz and Cz (Fz − 1.4 μV ± 1.0; Cz − 1.1 μV ± 0.7), and at M1 and M2 (M1 2.6 μV ± 1.1; M2 2.5 μV ± 1.2) was globally in the norm for age ± 2 SD.

### Genotype–phenotype correlations

Among clinical variables, deletion size was significantly correlated with expressive (*R* = − 0.67; *N* = 17; *p* = 0.003**) and receptive (*R* = − 0.71 N = 17; *p* = 0.002**) language disability scores. These results remained significant after Bonferroni correction.

Deletion size was not significantly correlated with N250 or MMN latencies and amplitudes regardless of the derivation. Nor was it correlated with pupil dilation at the sight of face and object.

## Discussion

A specific clinical and neurophysiological profile was identified for the first time in patients carrying the 22q13 deletion. This profile is characterized by an association of intellectual disability and language disability, which may lead to autistic behaviours, mainly in the first dimension of autism (i.e., communication impairment). Furthermore, neurophysiological explorations have highlighted an atypical pattern of gaze exploration that differs from the one previously reported in autistic populations. This pattern tends more towards a general lack of attention. Finally, the proposed electrophysiological endophenotype related to intolerance to change (i.e., shortened latency of MMN)^11^ was not observed, whereas an atypical N250 latency reinforces the hypothesis of abnormal auditory cortex maturation.

Clinical profile was mainly characterized by intellectual disability and language disability of frequency and severity comparable to those reported in the literature^24^. One study by Soorya et al.^1^ included careful clinical evaluation, caregiver reports and structured direct observation with ASD-specific diagnostic tools. Our results are consistent with this study, with most patients meeting ADI criteria for ASD (56 and 60% respectively). When both ADI and ADOS criteria were required, 39% of patients met all criteria in our study, mainly due to abnormal communication. Comparison with the study by Soorya et al.^1^ is not relevant as these authors mixed patients with full criteria and patients with only two ADI dimensions out of three. In DSM5, the “aloneness” social communication dimension has to be coupled with a second “sameness” dimension, which concerns reaction to change and repetitive, restricted behaviours to meet criteria for autism diagnosis. This association of two main dimensions corresponds to the original description by Kanner^25^. Furthermore, in this last version of the DSM^26^, distinction has been made between communication disorders and ASD, attributing a major role to the second dimension of autism.

Using a RRB scale, we were able to show that in patients with the 22q13 terminal deletion, the “sameness” dimension is globally missing. There is some evidence to suggest that there are two RRB ‘subtypes’. Sensorimotor RRBs and RRBs characterized by resistance to change, also known as repetitive sensorimotor actions (RSMA) and insistence on sameness (IS), respectively. RSMA are more likely to improve over time^27,28^ and are more strongly associated with cognitive ability than IS^29^. Consequently, IS has been shown to be particularly relevant for reflecting the second dimension^30,31^. However, this factor corresponds to items, which do not appear in the diagnostic algorithm of ADI-R. In the ADOS severity score, no minimal cut-off for RRB is required. This highlights the relevance of using an RRB-specific scale to assess the second dimension of autism. Finally, if these patients have autistic traits, in the vast majority of cases, they do not meet the current DSM5 criteria for diagnosis of ASD.

The neurophysiological profile found for patients with 22q13 deletion differs from the norm and differs from previous pattern published with the same protocol in ASD patients^11,14^.

Shortened MMN latency is a candidate endophenotype for autism, correlated with core symptoms. Indeed, bioclinical correlation between MMN latency and intolerance to change has been reported^11^. It also distinguishes autism from intellectual disability^12^. In our sample, MMN latency did not differ from the norm expected for age regardless of ADI and ADOS scores. Furthermore, we showed that patients with 22q13 deletion syndrome meeting ADI and ADOS criteria for autism tended to have longer latencies of N250 pattern than normal. The N250 auditory ERP component is known to reflect cortical auditory maturation. Furthermore, this component has been related to language abilities in children^13^. This electrophysiological pattern (i.e., normal MMN latency, larger N250 latency) may highlight an atypical development of cortical networks underlying language abilities in PMS patients, different from the one observed in autism.

In eye tracking, the mean time of screen exploration^32^ during visual screening of face and object^14^, reported to be altered in persons with autism, was preserved in this sample. Pupil reactivity, however, revealed an atypical physiological response (i.e., higher dilation) to object in 22q13 patients compared to control. The 22q13 deletion eye-tracking pattern (preserved exploration with atypical pupil reactivity) differed from the pattern of control and ASD patient (altered exploration with reduced pupil reactivity)^14^. This neurophysiological pattern, like electrophysiological patterns, suggests that there are different cortical networks underlying the semiology of PMS and autism.

Regarding size deletion, the only genotype–phenotype correlation in 22q13 syndrome was related to language disability, whereas no correlation was find with DQ. This result is in favour of the implication of *SHANK3* contiguous genes in language development. However, given the presence of mild to severe intellectual disability in the patients of this sample, this hypothesis has to be toned down. Four patients carried an identical deletion, with twins among them. This small deletion of 71 kb encompasses almost only the *SHANK3* gene. Twins had similar behavioural and electrophysiological profiles whereas the other two patients had very different clinical and electrophysiological profiles.

Language disability combined with cognitive deficits may lead to behavioural autistic symptoms but with different neurophysiological networks compared to typical autism. Altogether, these results highlight the extraordinary complexity of an atypical network which may lead to autistic behaviours. Furthermore, it reminds us that not only social communication problems are required to make an autism diagnosis. This may explain that in the past decade, patients with communication difficulties may have had an overrated autism diagnosis. A clarification of underlying mechanisms is essential to guide therapeutic intervention. In this study, it may indicate that intensive and early speech therapy is more relevant for PMS patients than a specific autistic behavioural programme.
