# Author Correction: 22q13 deletion syndrome: communication disorder or autism? Evidence from a specific clinical and neurophysiological phenotype

**DOI:** 10.1038/s41398-019-0435-4

**Published:** 2019-02-28

**Authors:** Laura Ponson, Marie Gomot, Romuald Blanc, Catherine Barthelemy, Sylvie Roux, Arnold Munnich, Serge Romana, Nadia Aguillon-Hernandez, Valérie Malan, Frédérique Bonnet-Brilhault

**Affiliations:** 1 UMR 1253, iBrain, Université de Tours, Inserm, Tours France; 2 Centre Universitaire de Pédopsychiatrie, CHRU de Tours, Tours, France; 3 Université Paris Descartes, Sorbonne Paris Cité, Institut de Psychologie, Laboratoire de Psychopathologie et Processus de Santé (EA 4057), Paris, France; 4 Université Paris Descartes, Sorbonne Paris Cité, Paris, France; 5 Laboratory of Molecular and Pathophysiological Bases of Cognitive Disorders, INSERM UMR 1163, Imagine Institute, Necker-Enfants Malades Hospital, Paris, France; 6 Service d’Histologie-Embryologie et Cytogénétique, Hôpital Necker-Enfants Malades, AP-HP, Paris, France

**Correction to:** *Translational Psychiatry* (2018) **8**: 146;

10.1038/s41398-018-0212-9; Published online 8 August 2018

Since the online publication of the above article, the authors have noted errors with the author list. The author names were listed as ‘(last name)(first name)’ instead of ‘(first name)(last name)’.

The authors and publisher apologise for any inconvenience caused by this error.

The correct author list is:

Laura Ponson, Marie Gomot, Romuald Blanc, Catherine Barthelemy, Sylvie Rouch, Arnold Munnich, Serge Romana, Nadia Aguillon-Hernandez, Valérie Malan and Frédérique Bonnet-Brillhault.

This has now been corrected in both the original PDF and HTML versions of the article.

